# Periodontitis and Systemic Disease: Association or Causality?

**DOI:** 10.1007/s40496-017-0121-7

**Published:** 2017-01-23

**Authors:** Lewis Winning, Gerard J. Linden

**Affiliations:** 1 Centre for Public Health, School of Medicine Dentistry and Biomedical Sciences, Queen’s University Belfast, Belfast, UK; 2 School of Dentistry, Queen’s University, Grosvenor Road, Belfast, BT12 6BP UK

**Keywords:** Diabetes, Obesity, Metabolic syndrome, Periodontitis, Mendelian randomisation

## Abstract

**Purpose of Review:**

The aim was to assess recent evidence that diabetes, metabolic syndrome (MetS) and obesity impact the progression of periodontitis.

**Recent Findings:**

Electronic searches using Embase, Medline, and Web of Science were carried out for epidemiological studies on humans, published between 2014 and 2016. A small number of prospective studies and systematic reviews were identified that in general provide further support for the hypothesis that diabetes, metabolic syndrome and obesity can adversely affect the periodontal condition.

**Summary:**

Confounding remains the most challenging issue in the interpretation of the associations found between diabetes, MetS, obesity and periodontal disease. Recent research applying a Mendelian randomisation approach concluded that the association between obesity and periodontitis is confounded and questioned a role for obesity in causation. Further studies are warranted to assess the issue of causality.

## Introduction

The periodontitis-systemic disease relationship constitutes an important part of clinical periodontal research. Research activity has grown continuously since the late 1980s with about one third of all recent periodontal studies now dealing with the relationship between periodontal disease and systemic disease [1•]. Further to this, a total of 57 different systemic disease conditions are now being researched in relation to periodontal diseases [2].

The periodontitis-systemic disease relationship is commonly described as being ‘*two-way*’ or ‘*bi-directional’*. However, the design of many observational epidemiological studies does not allow directionality to be firmly established and so by default, any associations identified will be *bi-directional* until data emerge that provide clarification. Currently, we do not have a full understanding of the importance of the numerous associations reported and in particular whether they play a part in causation. The conclusion of an expert panel at the joint European Federation of Periodontology/American Association of Periodontology workshop on ‘Periodontitis and Systemic Diseases’ was that ‘*reported associations do not imply causality and the establishment of causality would require new studies that fulfil the Bradford Hill or equivalent criteria*’ [3].

We have reviewed recent work published since 2014, focusing on prospective studies and systematic reviews where these exist, to examine whether progress has been made. The review is focused on systemic diseases or conditions that could impact the development or progression of periodontitis. Given the constraints of the large number of possible candidates [2], we have limited the focus of this review to diabetes, metabolic syndrome and obesity as they have been suggested to have a causative role in periodontitis. Discussion of the biological mechanisms through which these exemplars could increase the risk of periodontitis is beyond the scope of this review. A further paper in this edition has reviewed studies of periodontitis as a possible risk factor for systemic diseases.

## Diabetes

Diabetes mellitus comprises a group of metabolic diseases in which the characteristic phenotype is loss of control of glucose homeostasis, resulting from defects in insulin secretion, insulin action or both [4]. In 2015, there were an estimated 415 million people worldwide with diabetes and this is projected to rise to 642 million by 2040 [5]. In 2015, more than 10% of adults in 25 states of the USA had diabetes [6].

### Baseline

It is widely accepted that there is a bi-directional association between periodontitis and diabetes [7]. It is well documented that periodontal disease is more prevalent and severe in those with diabetes, but most studies supporting this observation have been cross-sectional precluding the ability to firmly demonstrate the direction of the association [8]. However, there have been prospective studies and a good baseline for the current review is a large study in the USA which suggested diabetes control, but not aetiology (type 1 or type 2 diabetes), was associated with accelerated periodontal attachment loss progression [9]. This was reinforced by Taylor and colleagues [10], who suggested it may be the level of metabolic control and duration of diabetes that influence periodontal disease risk, with a significant heterogeneity among diabetic individuals.

### Recent Studies

A recent prospective study investigated the influence of glycaemic control on the progression of periodontitis during periodontal maintenance therapy (PMT) [11]. Type 2 diabetes mellitus (T2DM) control was monitored by HbA1c percentage over a 5-year period. Participants matched for sex and smoking were divided into three groups: 23 with diabetes and poor glycaemic control (PGC), 23 with diabetes and good glycaemic control (GGC), and 46 controls with no diabetes (NDC). Progression of periodontitis was defined as an increase of interproximal clinical attachment loss of ≥3 mm in at least two teeth. Progression of periodontitis was significantly higher in the PGC compared to GGC and NDC participants. Multiple logistic regressions showed that for progression of periodontitis, a HbA1c ≥6.5% had an odds ratio (OR) of 2.9 (95% CI 1.43–9.81). A limitation was the small group sizes which prevented stratification for the effect of smoking. There was adjustment for smoking in the analysis; however, it was not clear if there was an interaction between smoking and glycaemic control. Despite this, and the restricted population that the subjects were recruited from, the study did demonstrate that poor glycaemic control significantly predicted risk for the progression of periodontal destruction in those previously diagnosed with and treated for periodontitis.

A prospective study of 4033 Taiwanese subjects [12], aged 35–44, with no evidence of periodontitis, defined by a Community Periodontal Index (CPI) score <3 [13], had their fasting plasma glucose (FPG) measured at baseline. The number of newly diagnosed periodontitis cases (CPI score ≥ 3) at re-examination after 5 years of follow-up was 1129 (28.0%) normal FPG, 96 (32.3%) pre-diabetes, and 22 (38.6%) with T2DM at baseline. Multivariable analysis using Cox’s proportional hazard regression model showed an increased risk of incident periodontitis for subjects with pre-diabetes (hazard ratio (HR) = 1.25 (95% CI 1.00–1.57)) and T2DM (HR = 1.95 (95% CI 1.22–3.13)) after adjustment for all potential confounders. A novel finding of this study was the significant effect of pre-diabetes on incident periodontitis [12]. A limitation of the study was the use of the CPI score to identify periodontitis [14]. Further weaknesses were lack of information regarding the management of the hyperglycaemia and its possible effects.

Based on the available evidence to date, it seems likely that the level of metabolic control influences future periodontal disease risk. However, further longitudinal studies are warranted to assess what effect improved glycaemic control has on the progression of periodontitis. The evidence examining the influence of pre-diabetes as a condition that can potentially adversely affect periodontal condition is inconclusive and also requires further investigation as evidence can both be found for [12] and against [15].

## Metabolic Syndrome

Metabolic syndrome (MetS) relates to a cluster of disorders including excess body fat around the waist and abdominal area, increased blood pressure, elevated plasma glucose, elevated serum triglycerides and reduced serum high-density lipoprotein [16]. The most recent definition from the International Diabetes Federation is the presence of central obesity, defined as a waist circumference exceeding ethnic specific guidelines, plus two from four of the other factors [17]. It has been highlighted that 25% of the world’s adults have MetS and as such, they have a fivefold greater risk of developing T2DM [17].

### Baseline

The baseline for MetS and periodontitis is the systematic review and meta-analysis completed by Nibali and colleagues which used data from 19 cross-sectional and only one longitudinal study [18]. MetS was associated with the presence of periodontitis with an OR of 1.71 (95% CI 1.42–2.03); however, there was evidence of heterogeneity.

### Recent Studies

A further systematic review reported a significant number of studies demonstrating a positive association between MetS and periodontitis [19]. However, the authors did highlight that virtually, all evidence for the association was based on cross-sectional studies and throughout, there was considerable variation both in the measures of periodontal disease and the definitions of MetS [19].

The impact of MetS on the progression of periodontal disease was investigated in 125 older adults from Japan [20]. Development of periodontal disease was equated with ≥2 teeth demonstrating a longitudinal loss of proximal periodontal attachment of ≥3 mm over 3 years of follow-up. At baseline, 27 (21.6%) of the participants were determined to have MetS. Subjects with baseline MetS were 2.6 (95% CI 1.17–5.67) times more likely to develop periodontitis during the observation period after adjustment for known confounders. Limitations of the study included the small sample size, the length of follow-up and also the lack of detail with regard to the management of MetS and whether changes in components of this may have had an impact in terms of periodontal disease development.

A further prospective study investigated 760 men in the Department of Veterans Affairs Dental Longitudinal Study and Normative Ageing Study who were followed for up to 33 years in the USA [21•]. The men’s oral health, weight, medical health and lifestyle were monitored approximately every 3 years, meaning that instead of just baseline values, time-dependent repeated measurements of MetS components were available. Periodontal outcome events on each tooth were defined as progression to predefined threshold levels of probing pocket depth (≥5 mm), clinical attachment loss (≥5 mm) and evidence of radiographic alveolar bone loss (≥40% of the distance from the cementoenamel junction to the root apex). The statistical models, used to estimate the effect of MetS for periodontitis events, took account of both clustering of teeth within individuals and the time-dependent status of metabolic syndrome. MetS increased the hazard ratios for pocket depth ≥5 mm (HR = 1.37 (95% CI 1.14–1.65)), clinical attachment loss ≥5 mm (HR = 1.19 (95% CI 1.00–1.41)) and alveolar bone loss ≥40% (HR = 1.25 (95% CI 1.00–1.56)). The number of positive metabolic syndrome factors was also associated with each of these outcomes. The prospective design and extended follow-up period provides certain robustness to the observed association that MetS predicts worsening periodontal disease in men. A limitation is the study population, who were predominantly non-Hispanic white males in a small area of the USA, and there is therefore limited external validity.

## Obesity

Obesity has been defined as a systemic disease characterized by excessive body fat accumulation that can lead to adverse impacts on health conditions [22]. The World Health Organization reported about 13% of the world’s adult population (11% of men and 15% of women) were obese in 2014 [23]. The worldwide prevalence of obesity more than doubled between 1980 and 2014 [23]. This increase in global prevalence has been more notable in certain developed countries, such as the UK, where obesity prevalence has increased from 15% in 1993 to 26% in 2014 [24]. In the USA, adult obesity rates now exceed 35% in four states, 30% in 25 states and are above 20% in all states [25].

### Baseline

Systematic reviews with meta-analyses have identified modest positive associations between obesity and periodontitis with respective OR = 1.35 (95% CI 1.23–1.47) [26], and OR = 1.81 (95%CI 1.42–2.30) [27], being reported. A long-term prospective study in the USA of non-Hispanic white men with robust criteria for periodontitis found that obesity and central adiposity were associated with an increased risk of the progression of periodontal disease [28]. A large prospective study of 36,910 healthcare professionals also reported a significant association between obesity and self-reported periodontitis [29].

### Recent Studies

A recent systematic review only considered prospective longitudinal studies assessing the association between weight gain and the incidence of periodontitis [30]. Five studies fulfilled the inclusion criteria and were appropriate for meta-analysis. The component studies were conducted in high-income countries including one study in Finland, two in Japan and two in the USA. The combined collective sample size was 42,198 individuals. The meta-analysis showed that subjects who became overweight had an increased relative risk of 1.13 (95% CI 1.06–1.20) of developing periodontitis compared with counterparts who stayed normal weight. For those who became obese, the relative risk was higher at 1.33 (95% CI 1.21–1.47). The results do provide a clear positive association between weight gain and new cases of periodontitis. However, the authors did point out that this conclusion was based on limited evidence and highlighted the need for further longitudinal prospective studies in low- and middle-income countries.

One prospective study investigated the effect of obesity on periodontal attachment loss (PAL) progression in an urban population from south Brazil [31]. Progression of periodontitis was defined as proximal PAL of ≥3 mm in ≥4 teeth over 5 years of follow-up in 582 (333 males/249 females) participants. Females who were obese at baseline were at statistically significant higher risk of periodontitis progression than those who were normal weight with a relative risk of 1.64 (95% CI 1.11–2.43). No association was observed for males with a relative risk of 1.13 (95% CI 0.75–1.69). This suggests obesity in this population from South America was a risk factor for PAL progression in females but not in males. A major limitation was the high dropout rate. At baseline, 1568 subjects were screened but only 755 re-attended at 5 years, of which 582 met the inclusion criteria for analysis in this study. A further limitation was the lack of diabetes assessments. This is particularly important in obesity studies as it is well established that diabetes is associated with both obesity and periodontitis. Therefore, diabetes could have been a confounder of the association reported between obesity and periodontitis [32].

## Association or Causation

What overall interpretation can be placed on the studies to date? Many observational studies have reported associations which could reflect causal involvement of an exposure in the aetiology of a disease or condition that subsequently could not be confirmed by large-scale prospective randomized controlled trials (RCTs) [33•]. One classical example is the finding from longitudinal studies that hormone replacement therapy (HRT) led to a reduction in the risk for coronary heart disease (CHD). The obvious conclusion was that HRT had a protective effect; however, when prospective RCTs were completed, it became evident that HRT was actually responsible for an increased risk of CHD [34•]. This was explained by the ‘healthy-user effect’ whereby women who took HRT tended to be better educated, more aware of their health, likely to take other preventive advice, to be less likely to smoke, etc. These factors therefore confounded the association, and this is likely to have affected many observational studies resulting in the identification of spurious associations [35]. A major weakness of observational studies is that there is no protection against confounding. Most studies use statistical techniques to adjust for obvious confounders; however, there may be residual confounding reflecting the difficulty in controlling for all dimensions of relevant factors [33•]. This is a particular problem for studies investigating exposures such as obesity and diseases such as periodontitis which can be affected by factors which are difficult to measure such as behaviour in social networks [36], socioeconomic status and health service utilization [33•]. In addition, there is no protection against unknown or unrecognized confounders.

If we accept that confounding is a challenging issue, how can we test whether a disease or condition such as obesity is a causative factor for periodontitis? One way to control for confounding is to apply a randomized design. RCTs are used to test the effectiveness of interventions and are the gold standard for validating a particular method of treatment. From ethical and practical viewpoints, it is difficult to envisage an RCT with a test group who are provided with a mechanism to become obese and a control group who maintain normal weight. There are few conclusions on the natural history of disease that are supported by prospective RCTs [37]. The best that might be achieved are prospective cohort studies looking at incident cases; however, these are also liable to bias and confounding.

Mendelian randomization (MR), a method which exploits the random assignment of genes from parents to their children at meiosis, could provide a solution [33•]. In MR, a genetic variant associated with a phenotype is used as an instrumental variable (IV) to evaluate the causal relationship between that phenotype and the outcome of interest. The fundamental consideration is that the IV is regarded as independent of confounders [38•]. Therefore, if we can identify a gene variant which is closely linked to a putative risk factor, then we may be able to use MR to assess whether it is a true causal aetiological factor. In this context, variants in a number of genes have been shown to be associated with obesity [39, 40]. Subsequently, MR has been used to show that greater adiposity leads to higher CRP levels with no evidence of any reverse pathway [41], and that higher body mass index (BMI) has a causal relationship with cardiometabolic traits including heart failure, metabolic syndrome and type 2 diabetes [38•].

The Gene Lifestyle Interactions and Dental Endpoint (GLIDE) consortium applied MR to test whether there was a causal relationship between obesity and periodontitis [42•]. GLIDE had access to BMI, periodontal status and relevant genotype data from 13 studies of up to 68,761 individuals. Genotypes at FTO (rs1121980), MC4R (rs17782313) and TMEM18 (rs6548238) which represent the largest known effect sizes for BMI in European populations were used to provide a genetic risk score (GRS). Definitions of periodontitis were those applied in each of the participating cohorts. Higher BMI was significantly associated to periodontitis after adjustment for known confounders in subjects (*n* = 29,459) who had been clinically assessed for periodontitis (odds ratio 1.13 (95% CI 1.10–1.17). In obese compared with normal weight participants, the relationship was stronger OR = 1.33 (95% CI 1.18–1.50). The genotypes used were associated with BMI with a statistically significant trend in mean BMI per GRS unit. However, when BMI-associated genotypes were studied in relation to periodontitis, there were no significant associations in those with clinically assessed periodontitis (10,972 cases, 9681 controls) with the pooled OR = 1.00 (95% CI 0.97–1.03) per risk allele. The authors concluded that their MR analysis did not support a role for total adiposity as a causal risk factor for periodontitis [42•].

We completed a further MR analysis on 1271 dentate men in Northern Ireland who were participating in the PRIME study (for details of participants, see [43]). The adiposity-associated variant rs17782313 in melanocortin-4 receptor (MC4R) was used as an instrumental variable for waist circumference (WC) in an MR design. There was a significant association between variants in rs17782313 and WC (*p* = 0.01). Increased abdominal adiposity was significantly associated (*p* = 0.007) with increased periodontal attachment loss (PAL) which was used to measure periodontitis. However, when the instrumental variable was applied to investigate the effect of WC on PAL, the association was not significant. This further independent study using an MR approach did not support a causal relationship between abdominal adiposity and periodontitis. The results of these recent MR studies suggest that the epidemiological association between obesity and periodontitis may be confounded. The possibility of reverse causality, i.e. in the direction from periodontitis to obesity cannot be excluded by the MR studies described. Further speculation on periodontitis as a possible causative factor for obesity is beyond the scope of this review.

## Conclusion

Evidence from many observational studies, including an increasing number of prospective studies, supports associations between the conditions described (diabetes, MetS, obesity) with periodontitis. There is a need for balanced professional judgement of how meaningful these associations are. In this context, one recurrent problem is the substantial variations in the definitions used to identify periodontitis with few studies that meet stringent criteria for periodontitis [32]. Confounding remains the most challenging issue in the interpretation of the associations found between various health states such as those discussed in this review (diabetes, MetS, obesity) and periodontal disease. However, where it has been possible to interrogate one possible association, in the absence of confounding, recent studies using an MR approach do not provide evidence that supports obesity as a causative factor for periodontitis in contradiction to the outcomes of observational studies [42•]. One limitation to this method is that it can only be used where there are suitable functional polymorphisms, or markers linked to functional polymorphisms, that are relevant to the exposure of interest. However, it is possible that these may be identified; for example, there is increasing evidence that genetic variants are associated with T2DM [44]. Until further progress is made, we should accept that the associations reported may be important and periodontal diagnostic procedures should be routinely carried out in patients who present with diabetes, MetS and obesity.
